# Comparing CxBladder to Urine Cytology as Adjunct to Cystoscopy in Surveillance of Non-muscle Invasive Bladder Cancer—A Pilot Study

**DOI:** 10.3389/fsurg.2021.659292

**Published:** 2021-05-13

**Authors:** C. A. Chai, W. S. Yeoh, R. Rajandram, K. P. Aung, T. A. Ong, S. Kuppusamy, A. Nazran, K. Kumaran, A. H. A. Razack, J. Y. Teoh

**Affiliations:** ^1^Urology Division, Department of Surgery, Faculty of Medicine, University of Malaya, Kuala Lumpur, Malaysia; ^2^S.H. Ho Urology Centre, Department of Surgery, The Chinese University of Hong Kong, Hong Kong, China

**Keywords:** bladder cancer, CxBladder, cystoscopy, non-muscle invasive bladder cancer, urine cytology

## Abstract

**Purpose:** Guidelines advocate cystoscopy surveillance (CS) for non-muscle invasive bladder cancer (NMIBC) post-resection. However, cystoscopy is operator dependent and may miss upper tract lesions or carcinoma *in-situ* (CIS). Urine cytology is a common adjunct but lacks sensitivity and specificity in detecting recurrence. A new mRNA biomarker (CxBladder) was compared with urine cytology as an adjunct to cystoscopy in detecting a positive cystoscopy findings during surveillance cystoscopy in our center.

**Materials and Methods:** Consented patients older than 18, undergoing CS for NMIBC, provide paired urine samples for cytology and CxBladder test. Patients with positive cystoscopy findings would undergo re-Trans Urethral Resection of Bladder Tumor (TURBT).

**Results:** Thirty-five patients were enrolled from April to June 2019. Seven contaminated urine samples were excluded. The remaining cohort of 23 (82%) and 5 (18%) females had a mean age of 66.69 (36–89). Eight (29%) patients with positive cystoscopy finding underwent TURBT. All 8 patients also had positive CxBladder result. This shows that CxBladder has a sensitivity and negative predictive value (NPV) of 100%, specificity of 75% and positive predictive value (PPV) of 62% in predicting a positive cystoscopy finding. TURBT Histo-pathological findings showed Low-grade Ta NMIBC in one patient (4%), and 7 (25%) patients had inflammatory changes. Urine cytology was only positive in one patient with a positive cystoscopy finding. This led to a sensitivity of merely 13% and NPV of 74%, while specificity and PPV was 100% in predicting a positive cystoscopy finding.

**Conclusion:** CxBladder had high NPV and sensitivity which accurately predicted suspicious cystoscopy findings leading to further investigation. It has great potential for use as adjunct to cystoscopy for surveillance of NMIBC.

## Introduction

Bladder cancer is identified as the 11th most commonly diagnosed cancer in the world (1, 2); of which 75% of patients presented initially as non-muscle invasive bladder cancer (NMIBC) (3). The EORTC Genito-Urinary Cancer group reported that non-muscle invasive bladder cancer is associated with a high recurrence rate after transurethral resection of bladder cancer (TURBT) of up to 80% in 5 years (4). Hence, various international guidelines such as the European Association of Urology (EAU), National Comprehensive Cancer Network (NCCN) and American Urological Association (AUA) guidelines advocate close monitoring and routine surveillance with cystoscopy as the current standard of care (5–7).

However, cystoscopy is operator dependent and upper tract lesions, early tumor or carcinoma *in-situ* (CIS) may sometimes be inconspicuous. Some literature recommend employing cystoscopy advancements such as fluorescence cystoscopy or narrow-band imaging in an attempt to assist in the identification of bladder cancer recurrence (8, 9). Currently, the commonly used adjunct to cystoscopy in surveillance of bladder cancer is urine cytology, even though studies have shown it lacks sensitivity and specificity in detecting bladder cancer recurrence. Furthermore, urine cytology is usually more accurate in detecting high-grade urothelial carcinoma, as evidence has shown a very low sensitivity for low-grade tumors (10–12).

A novel commercially available urine-based test (CxBladder) using mRNA biomarkers to detect bladder cancer recurrence in urine samples has been developed. CxBladder urine biomarkers have strong differential expression between tumors and normal bladder tissue, and the ability to identify broad inter-tumor heterogeneity known to bladder cancer (13). Although CxBladder was reported to hold better potential than urine cytology as an adjunct to cystoscopy, there is still limited studies to clearly establish CxBladder's superiority (14); hence it is still not a standard test in clinical guidelines especially in this region.

Therefore, this pilot study aims to compare CxBladder with urine cytology as an adjunct to cystoscopy in predicting positive cystoscopy findings leading to subsequent TURBT in surveillance of NMIBC.

## Materials and Methods

### Design

This was a prospective, single-center cohort study. We recruited 35 patients from the University Malaya Medical Center, Kuala Lumpur, who underwent surveillance cystoscopies to rule-out recurrence of bladder cancer. The primary outcome is to predict a positive cystoscopy findings requiring subsequent TURBT using both CxBladder or Urine Cytology.

### Ethical Approval

Ethics approval was acquired from University Malaya Medical Center ethics board (No.: 2019129-7072). This study was carried out following the principles of the Declaration of Helsinki and International Conference on Harmonization Good Clinical Practice guidelines.

### Patients

Patients aged 18 years old or older undergoing surveillance cystoscopies after being diagnosed with primary or recurrent NMIBC during the past 2 years were eligible for this study. Upon consent, patients were given a 50 mls specimen container to provide a fresh voided midstream urine sample before undergoing cystoscopy. The collected urine was separated into paired urine samples and sent for urine cytology and the CxBladder test. Patients who had active urinary tract infections were excluded from this study.

### Assessment

The CxBladder assesses the probability of bladder cancer recurrence by extracting and quantifying 5 mRNA biomarkers which are believed to be in higher concentrations in urine sample of bladder cancer patients (13). The biomarkers genes identified are: MDK, HOXA13, CDC2, IGFBP5, CXCR2. This is achieved by reverse transcription (RT) quantification polymerase chain reaction (13).

Results acquired from this study were entered into prespecified data collection forms before analysis. The results from CxBladder test kits were mailed to the investigator in a registered sealed document courier service as well as in a password encrypted email to ensure patients' data confidentiality.

Patients with positive cystoscopy findings were counseled and subjected to re-TURBT and treatment as per clinical guidelines. Patients with negative cystoscopy findings but positive urine cytology and/or CxBladder would undergo a repeat cystoscopy examination at an earlier date with a CT scan to rule out extravesical recurrence.

### Statistical Analysis

The comparative performance of CxBladder and cytology was analyzed by calculating the negative predictive value (NPV) and positive predictive value (PPV) of each. Sensitivity and specificity were also calculated for comparative purposes. These performance metrics, Numerical were represented as median ± standard deviation and/or frequencies using Microsoft Excel. These performance metrics and their 95% confidence intervals (CI) were calculated by standard methods using an online calculator.^1^

## Results

### Patients Characteristic

**Figure 1:**
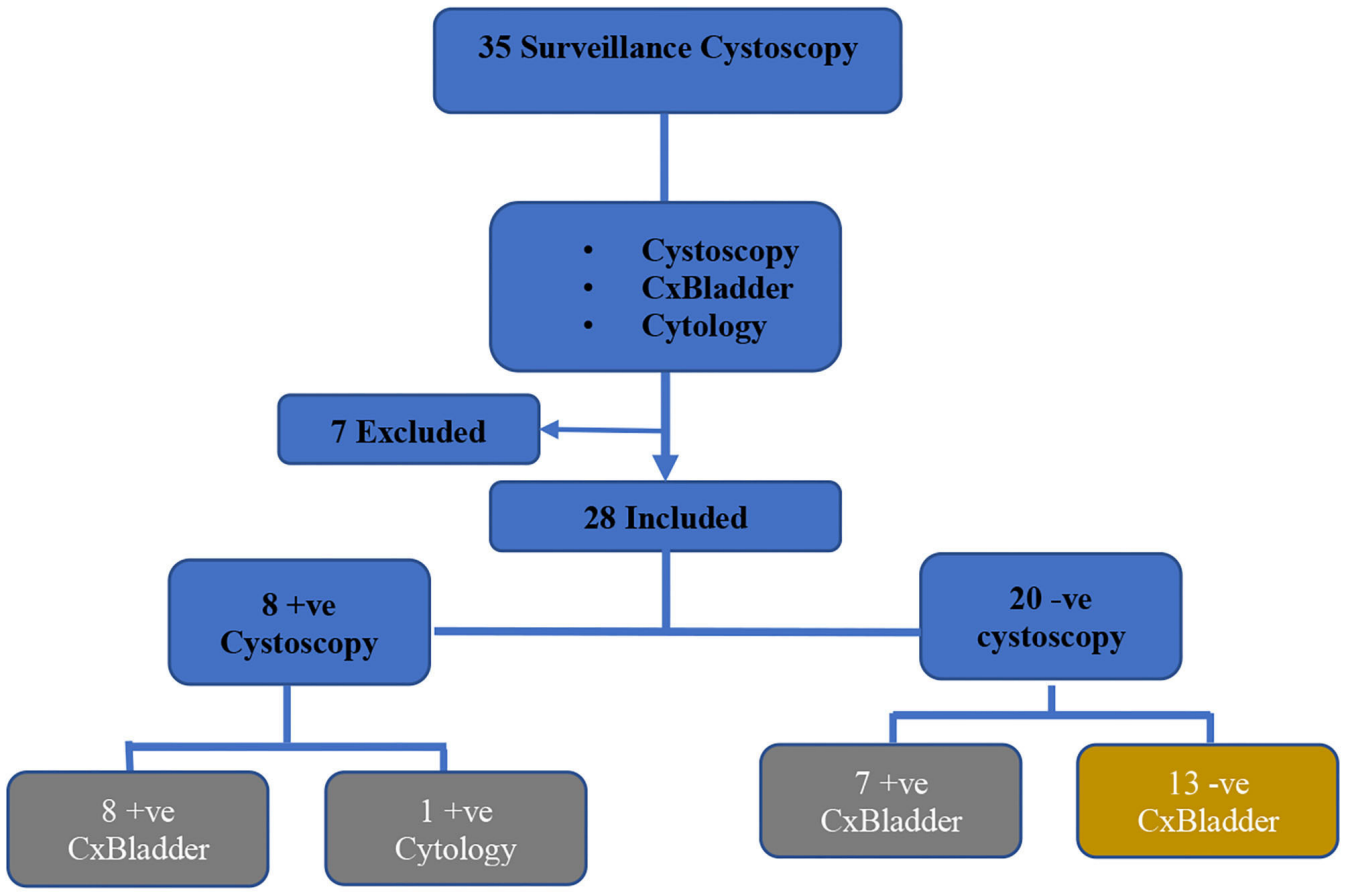
Consort diagram of the selection criteria of bladder cancer cases.

A total of 35 patients who were under follow up for bladder cancer surveillance consented for this study between April to June 2019. Seven were excluded from this study after urine samples sent were contaminated and deemed not suitable for CxBladder analysis as they were suggestive of urinary track infections or proteinuria (Figure 1).

Out of 28 remaining bladder cancer patients, 82% (*n* = 23) were males and 18% (*n* = 5) were females. The median age was 70-years-old (range: 59–74) (Table 1). The majority of the patients with non-muscle invasive bladder cancer consisted of Chinese ethnicity (61%, *n* = 17), followed by Indian (21%, *n* = 6), Malay (15%, *n* = 4) and another race (3%, *n* = 1). Sixty one percentage (*n* = 17) of these patients were smokers.

**Table 1:** Patient demographics of CxBladder surveillance.

	**No (%)**	**Positive CxBladder**	**Negative CxBladder**
**Median age** **+** **IQR**	70 (59–74)		
**Gender**			
Male	23 (82%)	12 (43%)	11 (39%)
Female	5 (18%)	3 (11%)	2 (7%)
**Race**			
Chinese	17 (61%)	9 (32%)	8 (29%)
Malay	4 (15%)	3 (12%)	1 (3%)
Indian	6 (21%)	2 (7%)	4 (14%)
Others	1 (3%)	1 (3%)	
**Risk**			
**Smoker**	17 (61%)	9 (32%)	8 (29%)
**Bladder Ca Staging**			
Ta	15 (54%)	8 (29%)	7 (25%)
T1	13 (46%)	7 (25%)	6 (21%)
**Bladder Ca grading**			
High grade	18 (64%)	8 (29%)	10 (35%)
Low grade	10 (36%)	7 (25%)	3 (11%)
**Bladder Ca risk group**			
Low risk	9 (32%)	4 (14%)	5 (18%)
Intermediate risk	7 (25%)	4 (14%)	3 (11%)
High risk	12 (43%)	7 (25%)	5 (18%)
**Previous intravesical therapy**			
Mitomycin	10 (35%)	4 (14%)	6 (21%)
BCG	15 (54%)	8 (29%)	7 (25%)
N/A	3 (11%)	3 (11%)	

### Tumor Characteristic

Examination of the tumor characteristic (T-staging) showed almost equal distribution of Ta (*n* = 15) and T1 (*n* = 13). The grade of the lesion in our cohort showed predominantly high grade (*n* = 18) tumor compared to low grade (*n* = 10) disease. Distribution according to risk group shows 32% low risk disease (*n* = 9), 25% intermediate risk disease (*n* = 7) and 43% (*n* = 12) high risk disease (Table 1). Four patients had history of CIS in previous histopathological findings. Fifty four percentage (*n* = 15) of these patients received intravesical BCG while 35% (*n* = 10) received intravesical mitomycin during the course of treatment.

### Cystoscopy Finding

Twenty nine percentage (*n* = 8) of the bladder cancer patients had cystoscopy findings suspicious of recurrence and underwent TURBT. Of these, all patients had positive CxBladder findings. The histopathological findings showed 3% (*n* = 1) low-grade Ta recurrent bladder cancer, and 25% (*n* = 7) inflammatory changes. Three percent (*n* = 1) of patient in whom CxBladder test was positive, displayed positive urine cytology as well, but with a negative histopathological outcome (Figure 1). Among 71% (*n* = 20) patients with negative cystoscopy findings; 25% (*n* = 7) of them had a positive CxBladder result (Figure 1). These cases were scheduled for early repeat cystoscopy for reassessment before CT imaging to rule out extravesical recurrence.

CxBladder showed a very high sensitivity (100%) and negative predictive value (NPV) (100%) [95% CI = 2.86 (2.85714–2.85715)] in detecting a positive cystoscopy finding as opposed to urine cytology which showed a mere sensitivity of 13% and a negative predictive value of 74% (Table 2).

**Table 2:** CxBladder vs. urine cytology for bladder cancer cases.

	**CxBladder**	**Cytology**
Sensitivity	100%	13%
Specificity	75%	100%
Positive Predictive Value	62%	100%
Negative Predictive Value	100%	74%
95% C.I	2.86 (2.85714–2.85715)	

Conversely, urine cytology demonstrated a much higher specificity and PPV (100%) while CxBladder demonstrated much lower specificity (75%) and PPV (62%) in predicting a positive cystoscopy finding.

### Relations Between Previous Intravesical Mitomycin C/BCG and CxBladder

In our cohort, 35% (*n* = 10) of the patients received intravesical mitomycin for previous low-grade histology of bladder cancer. Of these, four patients (14%) showed positive CxBladder test while six (21%) were negative.

Similarly, for patients with high grade histology of bladder cancer who received prior intravesical BCG therapy; the result of CxBladder were fairly equally distributed with eight patients (29%) having positive CxBladder and seven (25%) displayed negative result.

## Discussion

This is a pilot study in our local center to access the feasibility of using CxBladder compared to urine cytology as an adjunct to white light cystoscopy for post-operative surveillance of non-muscle invasive bladder cancer. We compared the likelihood of each to predict a positive cystoscopy finding requiring TURBT. A histologically proven malignancy is the only way to confirm the diagnosis of recurrent bladder cancer, this can only be done by performing a TURBT in the event of a postitive cystoscopy finding (15). Hence, the ability to first predict a positive cystoscopy finding will have the potential to serve as a screening tool to “rule-out” the need for invasive cystoscopy examination.

Our study had shown that CxBladder had a significantly higher NPV (100%) [95% CI = 2.86(2.85714–2.85715)] in detecting bladder cancer recurrence. This coincides with previously reported studies which identified CxBladder as a superior “rule-out” test than urine cytology (14). In a study by Kavalieris et al. among 1036 patients, it was reported that CxBladder has a sensitivity of 93% and NPV of 97% in detecting bladder cancer recurrence. Therefore, in patients who are unable to tolerate a cystoscopy or demonstrated suspect compliance to regular cystoscopy surveillance, CxBladder may play a role as an initial surveillance tool to “rule out” before submitting the patient to cystoscopy which is currently the standard of care (5–7). This can also be especially useful in rare cases of patients with underlying spinal cord injury where flexible cystoscopy might lead to complications such as autonomic dysreflexia (16).

The potential use of CxBladder as a screening tool with high NPV could gain importance especially in the Covid-19 pandemic era. A non-invasive screening with CxBladder could reduce the unnecessary exposure of patients to health-care workers and environment which could subsequently reduce the risk of Covid-19 infection. Furthermore, reduction of cystoscopy procedures plus full personal protective equipment (PPE) use can potentially be more cost effective in the future.

Recently, a prospective randomized study has found that prior knowledge of positive urine cytology may improve the quality of follow-up cystoscopy (17). Madelon et al. described in their study that diagnostic review bias may increase scrutiny of cystoscopic examination during cystoscopy surveillance for NMIBC. Furthermore, Madelon et al. did not notice an increase in false positive cystoscopy findings in patients who had a positive urine test (17). Hence, supporting the adjunctive role of CxBladder performed before follow up surveillance cystoscopy.

In our cohort of patients, there were 25% (*n* = 7) false-positive results for CxBladder. These patients showed negative cystoscopy findings despite a positive CxBladder result.

We postulated that CxBladder may be very sensitive to inflammatory changes in the bladder. However, we cannot confirm whether this is indeed a false positive CxBladder result, or due to a high pickup of microsatellite recurrence where white light cystoscopy failed to pick up disease recurrence. Even though some guidelines advocate random biopsy for patients with discordance of positive urine cytology and negative cystoscopy findings, urine cytology results for these seven patients were also negative. Hence, we decided to avoid possible invasive complications and over treatment by arranging earlier cystoscopy appointment to ensure no tumor recurrence is missed.

The main limitation of our study is the small number of patients in our cohort. However, our results coincide with those of larger studies in demonstrating the high sensitivity and NPV of CxBladder in predicting a positive cystoscopy findings (14, 16). As this was a pilot study, the small number of bladder cancer patients was sufficient to prove that CxBladder is a feasible adjunct to cystoscopy in our local population. A larger cohort will be needed to further support our findings. Long-term follow-up as per standard guidelines for NMIBC will provide more robust data on the feasibility of employing CxBladder as the preferred adjunct compared to urine cytology in the near future.

## Conclusion

Cxbladder has high NPV and sensitivity in predicting the chances of a suspicious cystoscopy finding that will lead to further investigation. In-depth study using CxBladder on a larger cohort will be needed to justify these findings as it may offer less invasive surveillance for bladder cancer before proceeding with cystoscopy examination. Further investigation of patients who have negative cystoscopy findings with a positive CxBladder result will provide more data in assessing the sensitivity of CxBladder in picking up occult bladder cancer recurrence.

## Data Availability Statement

The original contributions presented for this study are included in the article/supplementary material, further inquiries can be directed to the corresponding author/s.

## Ethics Statement

The studies involving human participants were reviewed and approved by University Malaya Medical Center ethics board (No.: 2019129-7072). The patients/participants provided their written informed consent to participate in this study. Written informed consent was obtained from the individual(s) for the publication of any potentially identifiable images or data included in this article.

## Author Contributions

CAC wrote the manuscript, designed, and carried out this study. WSY, AN, and KPA performed the cystoscopy examinations, read, and approved the menuscript for submission. RR performed the statistical analysis, read, and approved the menuscript for submission. TA, SK, KK, and AR, read, edited, and approved the manuscript for submission. JT reviewed, edited, and approved the manuscript for submission. All authors contributed to the article and approved the submitted version.

## Conflict of Interest

The authors declare that the research was conducted in the absence of any commercial or financial relationships that could be construed as a potential conflict of interest.

## Footnotes

^1^https://www2.ccrb.cuhk.edu.hk/stat/confidence%20interval/Diagnostic%20Statistic.htm.
